# User-friendly platform for analysis of high mass intact proteins and glycopeptides by laser desorption/ionization-mass spectrometry based on copper oxide particles

**DOI:** 10.1007/s00216-023-05072-0

**Published:** 2023-12-08

**Authors:** Valeria Springer, Yuye Zhou, Ángela Y. Aguilera, Åsa Emmer

**Affiliations:** 1 INQUISUR - Departamento de Química, Universidad Nacional del Sur (UNS)-CONICET, B8000CPB, Bahía Blanca, Buenos Aires Argentina; 2 Department of Chemistry, Analytical Chemistry, School of Engineering Sciences in Chemistry, Biotechnology and Health, KTH Royal Institute of Technology, 100 44 Stockholm, Sweden

**Keywords:** Copper oxide particles, High mass proteins, Laser desorption/ionization, Mass spectrometry, Multipurpose platform

## Abstract

Matrix-assisted laser desorption/ionization-mass spectrometry (MALDI-MS) based on micro/nanostructured materials with different natures has received increasing attention for the analysis of a wide variety of analytes. However, up to now, only a few studies have shown the application of simple platforms in MALDI-MS for the identification of intact proteins. The present work reports on the application of copper oxide particles (Cu_2_O PS), obtained by a greener route, in combination with low amounts of 2,5-dihydroxybenzoic acid (DHB) as a novel hybrid platform. The combined Cu_2_O PS@DHB matrix, containing only 2.5 mg mL^−1^ of particles and 10 mg mL^−1^ of DHB, was easily applicable in MALDI-MS without surface modification of target plates. Under optimal conditions, the analysis of intact proteins up to 150,000 Da was possible, including immunoglobulin G, bovine serum albumin, and cytochrome C with adequate spot-to-spot signal reproducibility (RSD < 10%). In addition, the analysis of glycopeptides from IgG digests was carried out to prove the multipurpose application of the Cu_2_O PS@DHB platform in the low m/z range (2500–3000 Da). From the obtained results, it can be concluded that the optical and surface properties of as-synthesized Cu_2_O PS are likely to be responsible for the superior performance of Cu_2_O PS@DHB in comparison with conventional matrices. In this sense, the proposed user-friendly methodology opens up the prospect for possible implementation in bioanalysis and diagnostic research.

**Graphical Abstract:**

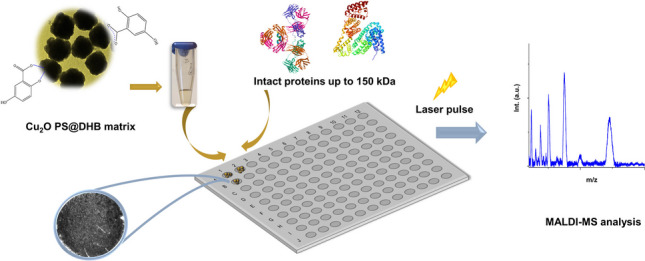

**Supplementary Information:**

The online version contains supplementary material available at 10.1007/s00216-023-05072-0.

## Introduction

Laser desorption/ionization (LDI) is a soft ionization technique that shows several distinctive characteristics such as satisfactory tolerance for salts or detergents present in the samples, low fragmentation, and the capability to analyze macromolecules, thus supporting its extensive application in the analysis of proteins by mass spectrometry (MS) [1]. When the ionization process is the result of laser radiation on a crystallized mixture of the sample and an organic matrix, it is called matrix-assisted laser desorption/ionization (MALDI). Hence, the matrix mediates the transfer of energy from a pulsed laser beam (e.g., Nd:YAG laser, *λ* = 355 nm) to the analyte molecules to assist its desorption and ionization. Conventional organic matrices have different optimal mass ranges for the analyte ionization, being necessary the combination of some of them to cover the whole mass range when identifying an unknown sample. For analysis of intact proteins, α-cyano-4-hydroxycinnamic acid (CHCA), sinapinic acid (SA), and 2,5-dihydroxybenzoic acid (DHB) are normally evaluated as matrices [2]. However, in most of the cases, the main drawback remains to be the inhomogeneity of the analyte/matrix co-crystallization on the target plate, which leads to shot-to-shot and spot-to-spot lack of reproducibility, generating poor mass resolution at > 60,000 Da and even poor repeatability. Furthermore, the ionization efficiency of organic matrices is often low for proteins, thus requiring high concentrations for co-crystallization with the sample [3].

In recent years, large endeavors have been undertaken to propose efficient platforms with superior signal sensitivity that could extend the application of conventional MALDI-MS. Concerning this, it is a meaningful challenge to explore new UV-absorbing solid materials, such as organic and inorganic micro- and nanoparticles, that could provide remarkable advantages for desorption/ionization of a wide range of bio-molecules (e.g., amino acids, lipids, peptides, proteins). These characteristics are supported by the structure of particles with dimensions lower than 500 nm, where physical properties such as thermal dissipation, optical absorption, energy confinement, and plasmon resonances are enhanced [4–7]. Moreover, micro- and nanostructure-based matrices allow a satisfactory spot-to-spot reproducibility because more homogeneous coverage of spots can be achieved [5]. Among the inorganic structures studied so far, metal and metal oxide particles mainly based on iron, gold, silver, bismuth, titanium, and copper have been tested to assist the LDI process [7–12]. In this sense, it is also possible to reduce the chemical background in the low mass region and the generation of multiple adducts with analytes, commonly evidenced with conventional matrices [13, 14]. Nevertheless, for analysis of proteins in the large mass range, only few works present advantages in terms of reproducibility and mass accuracy. A recent study proposed the use of titanium nitride (TiN) nanopillar arrays and thin films as substrates for desorption/ionization of various proteins without surface modification or pre-treatment with low background noise and no fragmentation [15]. This work represented a useful alternative to a previous HgTe nanoparticle platform which is of concern in terms of potential health effect [16]. However, fabrication of TiN nanopillars requires a deposition step on silicon wafers followed by thermal treatment, involving time and specific instrumentation. In the same way, a recent work proposed boron-doped carbon nanowalls, synthesized by microwave plasma-enhanced chemical vapor deposition, to be used for detection of cytochrome C [17]. Despite these works using different particles and hybrid micro/nanostructured substrates, the development of user-friendly platforms for desorption/ionization that exhibit uniform response for intact proteins of different basicity and weight is still demanded, especially when focusing on clinical diagnostics and pharmaceutical applications. To the best of the authors’ knowledge, the application of green synthesized copper oxide particles for LDI-MS analysis of high molecular weight proteins has not yet been explored.

In this work, the cooperative effect of copper oxide particles (Cu_2_O PS), synthesized by a fast and environmentally friendly method, was evaluated for analysis of intact proteins up to 150,000 Da. To this end, a hybrid organic–inorganic platform composed by low amounts of 2,5-dihydroxybenzoic acid (DHB) and Cu_2_O PS was prepared. This platform was applied for rapid identification of cytochrome C, albumin from bovine serum (BSA), and human immunoglobulin G (IgG) at low concentrations (fmol/spot). Additionally, direct analysis of IgG digests was also assessed to show the versatile application of the proposed material for potential application in proteomics and clinical research.

## Materials and methods

### Reagents

Copper sulfate (CuSO_4_.5H_2_O), d-( +)-glucose, and polyvinylpyrrolidone (PVP) were obtained from Cicarelli (Buenos Aires, Argentina). Sodium hydroxide was provided by Anedra (Buenos Aires, Argentina). MALDI matrix, 2,5-dihydroxybenzoic acid (DHB), was purchased from Bruker Daltonics Gmbh (Bremen, Germany). Immunoglobulin G from human serum (IgG, 56,834-25MG), albumin from bovine serum (BSA, A3059), cytochrome C from equine heart (Cyt C), acetonitrile (ACN), and trifluoroacetic acid (TFA) were purchased from Sigma-Aldrich (Stockholm, Sweden). All reagents were of analytical grade and used without further purification. Ultrapure water with a resistivity of 18.2 MΩ cm at 25 °C (Millipore Synergy® 185 system, Bedford, MA, USA) was used throughout the experiments. For analysis of glycopeptides, the IgG standard was subjected to trypsin digestion and the digested mixture had a final concentration of 1 mg mL^−1^ [18].

### Synthesis of Cu_2_O PS and general characterization

Copper oxide particles (Cu_2_O PS) with uniform size and polyhedral morphology were prepared according to the method reported by Aguilera et al. [19], which was optimized through the response surface methodology. Briefly, the reduction of Cu^2+^ (2.5 mM) in aqueous solution was performed in the presence of glucose (0.16 M) at pH 10.0. In addition, PVP (1%, wt v^−1^) was employed as the stabilizing agent for particle generation and controlled size. The volume was made up to 10 mL, and the mixture was poured in a polytetrafluoroethylene (PTFE) tube for microwave irradiation for 80 s at 560 W in a domestic oven. Aiming to prevent significant losses of the solvent due to evaporation, the tube was closed with a PTFE screw cap during synthesis, and afterwards it was cooled to room temperature. The resulting light brown-orange solid was separated by centrifugation, washed with ultrapure water three times to eliminate excess of unreacted compounds, and finally resuspended in ultrapure water. A 30 mg mL^−1^ Cu_2_O PS suspension was stored under light protection. At the beginning of each working day, the Cu_2_O PS suspension was sonicated for 2 min (50 kHz, Sonorex RK 100H ultrasonic bath) and then appropriate dilutions from this solution were performed in ultrapure water to prepare a 5 mg mL^−1^ Cu_2_O PS suspension.

The size and morphology of as-prepared Cu_2_O PS were evaluated by transmission electron microscopy (TEM) using a JEOL 100 CX II microscope operated at 100 kV. The crystal phase of the material was determined by powder X-ray diffraction (XRD) using a Philips PW 1710 diffractometer with Cu Kα radiation (*λ* = 1.54059 Å) and a graphite monochromator operated at 45 kV, 30 mA, and 25 °C at a scan rate of 2θ s^−1^. UV–Vis spectra were obtained on a Cary 60 spectrophotometer (Agilent Technologies, USA). Hydrodynamic size of particles was measured by dynamic light scattering (DLS) at 25 °C with a Malvern Nano ZS90 equipment and the zeta potential (ζ) was calculated with the Smoluchowski equation. Samples were prepared in 10^−2^ M NaCl as the supporting electrolyte for all measurements. FTIR analysis was performed to characterize the surface groups on the particles. Spectra were recorded with a FTIR-NIR Thermo Scientific Nicolet iS50 spectrophotometer with attenuated total reflectance (ATR), single-bounce accessory, and a diamond crystal (incidence angle of 42°) in the wavenumber range from 600 to 4000 cm^−1^ at a resolution of 4 cm^−1^ and 120 scans.

### MALDI-MS analysis

The evaluation of Cu_2_O PS as an ionization-assisting material was performed on a MALDI-TOF–MS instrument (UltrafleXtreme, Bruker Daltonics, Bremen, Germany) with a Smart-beam TM II laser (Nd:YAG) operated at 355 nm, and working in positive mode. DHB solution (20 mg mL^−1^) was prepared in ACN:0.1% TFA (30:70 v/v) mixture. A hybrid matrix (Cu_2_O PS@DHB) was prepared by mixing equal volumes of freshly prepared Cu_2_O PS aqueous suspensions and DHB solutions to get a final concentration of 2.5 mg mL^−1^ and 10 mg mL^−1^, respectively. This mixture was sonicated for 2 min. Then, 0.5 µL of Cu_2_O PS@DHB matrix was dropped on the target plate (MTP 384 polished steel, Bruker Daltonics) with a micropipette. After the sample plate was air dried, 0.5 µL of a protein standard solution (or protein digest) was added and left to dry prior to analysis. Intact proteins were analyzed using linear detection mode, while peptides were analyzed using reflector mode. The laser intensity was set to 80% of the total intensity and spots were irradiated with 7000 shots in a random walk pattern. For each analysis, 5 replicates were used. Data were acquired with the FlexControl software and processed with the Flex Analysis version 3.4 (Bruker Daltonics).

### Human and animal rights

All human and animal samples were commercial products. There are no conflicts with human or animal rights.

## Results and discussion

### Characterization of Cu_2_O PS

**Fig. 1:**
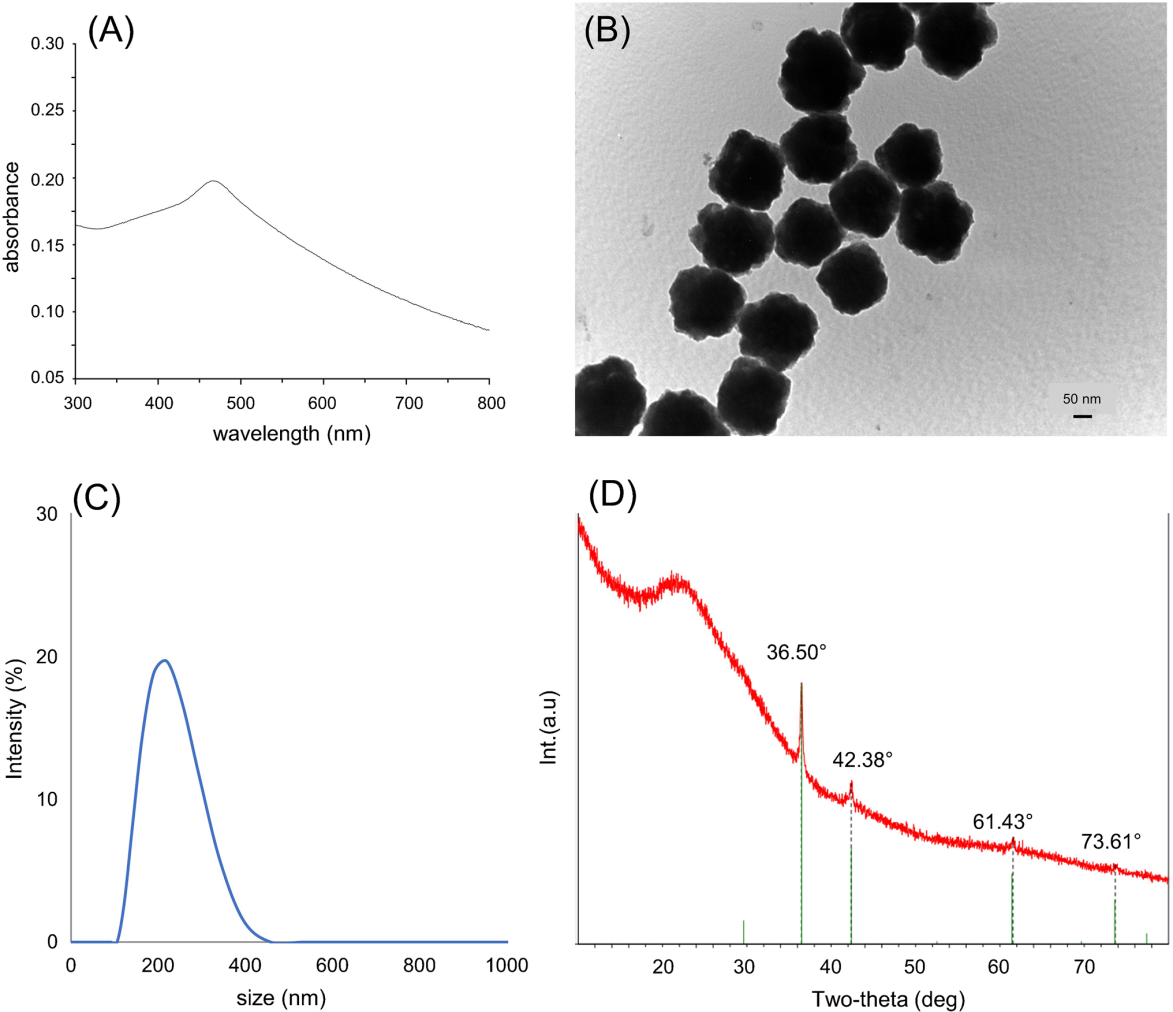
Characterization of Cu_2_O PS by (**A**) LSPR, λ_max_ = 470 nm, (**B**) transmission electron microscopy, TEM (100,000 ×), (**C**) dynamic light scattering (DLS), and (**D**) X-ray powder diffraction (XRD)

Synthesized Cu_2_O PS were initially characterized according to their optical properties by UV–Vis spectroscopy. As shown in Fig. 1A, the localized surface plasmon resonance (LSPR) band evidenced a maximum signal at 470 nm, which is associated to the plasma resonance excitation of copper atoms on the surface of particles. The LSPR intensity of Cu_2_O PS was also followed to evaluate the batch-to-batch reproducibility of the synthesis, with relative standard deviation (RSD%) values around 2.41% (*n* = 6). The size and morphology of particles were analyzed by TEM microscopy, showing a polyhedral morphology and an average size around 229 nm (Fig. 1B). Hydrodynamic size of Cu_2_O PS in aqueous suspension was determined by DLS (Fig. 1C), with an average value about 223 nm in agreement with that reported by TEM. In addition, the XRD analysis showed characteristic diffraction peaks at 36.50°, 42.38°, 61.43°, and 73.61° which correspond to the (111), (200), (220), and (311) planes, respectively (Fig. 1D). These peaks may be associated with face-centered cubic structures of Cu_2_O particles as recently confirmed in the literature [20].

### MS analysis based on Cu_2_O PS@DHB matrix

#### Evaluation of Cu_2_O PS for MALDI-MS

It is well known that the occurrence of inhomogeneous zones on the spots is one of the major drawbacks when using conventional organic matrices in MALDI-MS, which can lead to poor shot-to-shot and sample-to-sample reproducibility. In order to overcome this issue and the possible interference of the matrix itself, the use of diverse nanoparticles was recently proposed for analysis of small molecules [10, 13]. However, as capping agents are not generally included for these nanomaterials to avoid contamination from organic molecules, aggregation of particles, especially metals and metal oxides, can cause significant loss of ion signals. This is important for analysis of large molecules, including intact proteins, where a certain amount of an organic matrix could be still required to assist the ionization. Here, the performance of a hybrid matrix based on Cu_2_O PS and a minimum amount of DHB was tested. All experimental parameters were evaluated with IgG as a model protein considering its high molecular weight (~ 150 kDa).

An initial inspection of the co-crystallization of IgG with the matrix was performed (Fig. S1, Supplementary Material). A heterogeneous distribution of the organic matrix on the plate was observed, which can generate poor reproducibility between analyses, commonly observed in MALDI-MS. On the other hand, the addition of Cu_2_O PS led to a more homogeneous spreading on the surface of the spot with possible benefits in the co-crystallization process and minimization of “sweet spots” (Fig. S1B). Aiming to assure the use of Cu_2_O PS@DHB as matrix, MS spectra were obtained to verify the absence of extra signals when subjecting the matrix to irradiation, thus confirming the potential application of this hybrid platform in the mass range between 20,000 and 200,000 Da (Fig. S2).

**Fig. 2:**
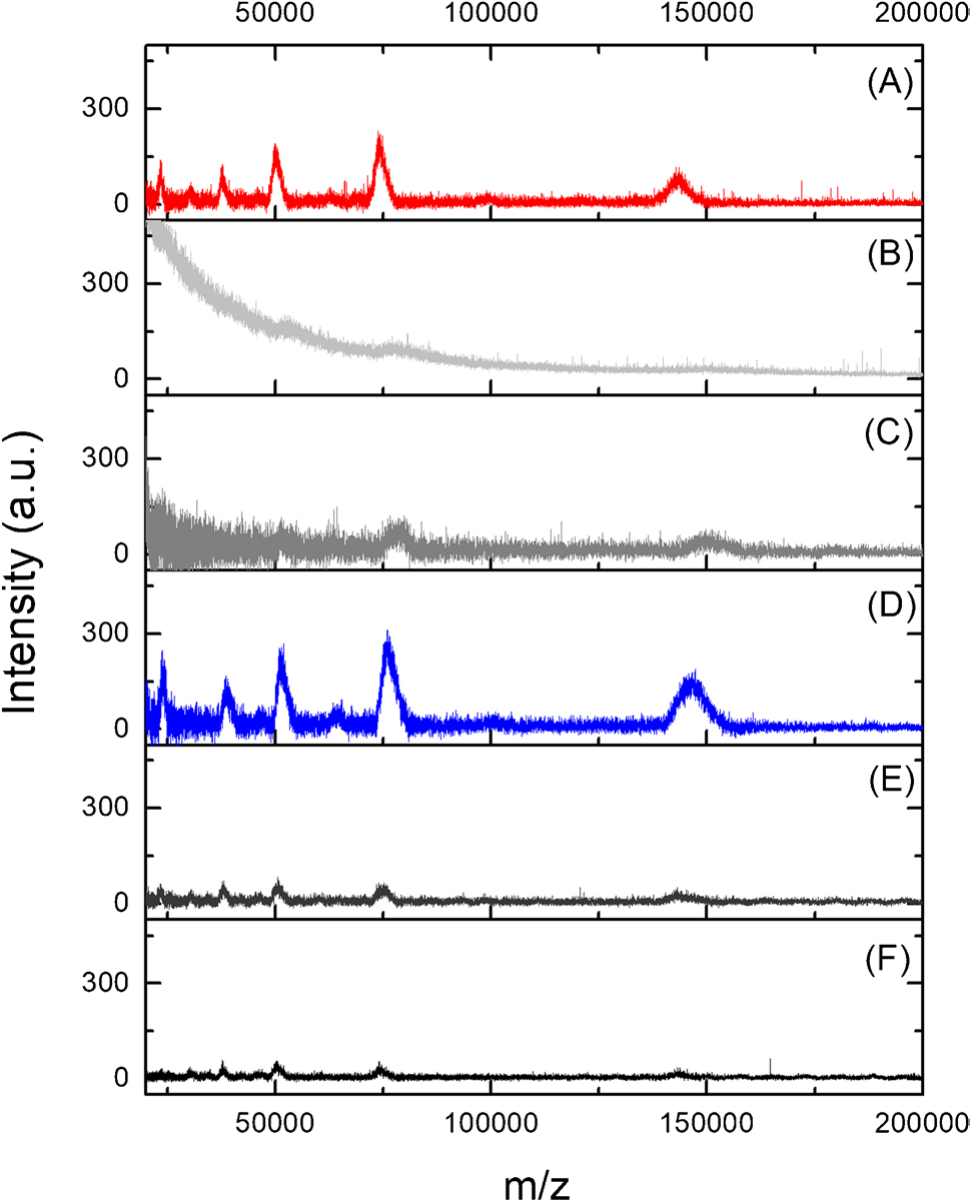
LDI**-**MS spectra of IgG (20 µg mL^−1^) measured in positive mode with the conventional matrix, 20 mg mL^−1^ DHB (**A**) and with matrices containing 10 mg mL^−1^ of DHB and different concentrations of Cu_2_O PS: (**B**) 10 mg mL^−1^, (**C**) 5.0 mg mL^−1^, (**D**) 2.5 mg mL^−1^, (**E**) 1.0 mg mL^−1^, and (**F**) 0.2 mg mL^−^^1^

Obtained MALDI-MS spectra of IgG when using the Cu_2_O PS@DHB and the comparison with the conventional DHB at two concentration levels are shown in Figure S3. Interestingly, the addition of Cu_2_O PS in the matrix produced an increase in the intensity of all analyte peaks. As an example, for the peak at m/z 75,000, the mean intensity increased from 591.0 ± 130.2 to 955.6 ± 96.4 when using the Cu_2_O PS@DHB, thus supporting the contribution of nanoparticles to the overall process. In this case, the synergistic effect between DHB and Cu_2_O PS might be associated to the adsorption of DHB molecules to the Cu_2_O PS in the mixture. Previous studies have demonstrated the binding of DHB to the surface of metal oxide nanoparticles (e.g., Fe_3_O_4_ and Al_2_O_3_) at low pH conditions. This interaction is mainly governed by a bidentate coordination boosted by the combination of carboxylic groups and the ortho-hydroxyl groups in the DHB molecule, which increase its electronic density [21, 22]. In this way, it is possible to achieve better stabilization of Cu_2_O PS in aqueous solution, supporting the more homogeneous distribution on the spots. In addition, a larger surface will be available for interaction with the protein molecules, and more energy may be transferred from the laser. Under these presumptions, different amounts of Cu_2_O PS in the matrix were evaluated aiming to get satisfactory MS signals. For this purpose, the concentration of particles was varied between 0.2 and 5.0 mg mL^−1^ by keeping the same volume ratio (1:1) with DHB solution (Fig. 2). An increase in the signal intensities was found when the matrix contained 2.5 mg mL^−1^ of particles in comparison to the conventional analysis, while a significant reduction was evidenced when the concentration was higher or lower than this value. In the first case, it is hypothesized that a low DHB/Cu_2_O PS ratio in the matrix leads to the generation of aggregates with an increase in the particle size, thus affecting the available surface area and dispersibility of the material on the spots, along with the loading capacity for analytes and transfer of energy for ionization. In fact, an intense background interference was experienced, while MS signals decreased severely when a concentration of Cu_2_O PS above 2.5 mg mL^−1^ was used, with the consequently low signal-to-noise response (Fig. 2B and C). On the other hand, a low concentration of particles may not be enough to assist the ionization process making its contribution negligible in the analysis (Fig. 2E and F). These findings suggested that a proper amount of both Cu_2_O PS and DHB should be used in the matrix to minimize the aggregation of particles, while providing a large surface area, satisfactory coverage, and homogeneous crystallization on spots. Hence, Cu_2_O PS@DHB matrix was prepared with 2.5 mg mL^−1^ of Cu_2_O PS while setting DHB concentration to 10 mg mL^−1^.

Moreover, the uniformity of Cu_2_O PS@DHB matrix coverage was evaluated by measuring the repeatability of signal intensities obtained under selected conditions. The resulting relative standard deviation (RSD%) was around 9.2% (*n* = 5), which corroborates that Cu_2_O PS synthesized in our laboratory in combination with low amounts of DHB produced a stable and homogeneous matrix, and allowed facile coverage of spots.

#### Effect of pH and laser power on the LDI-MS measurements

**Fig. 3:**
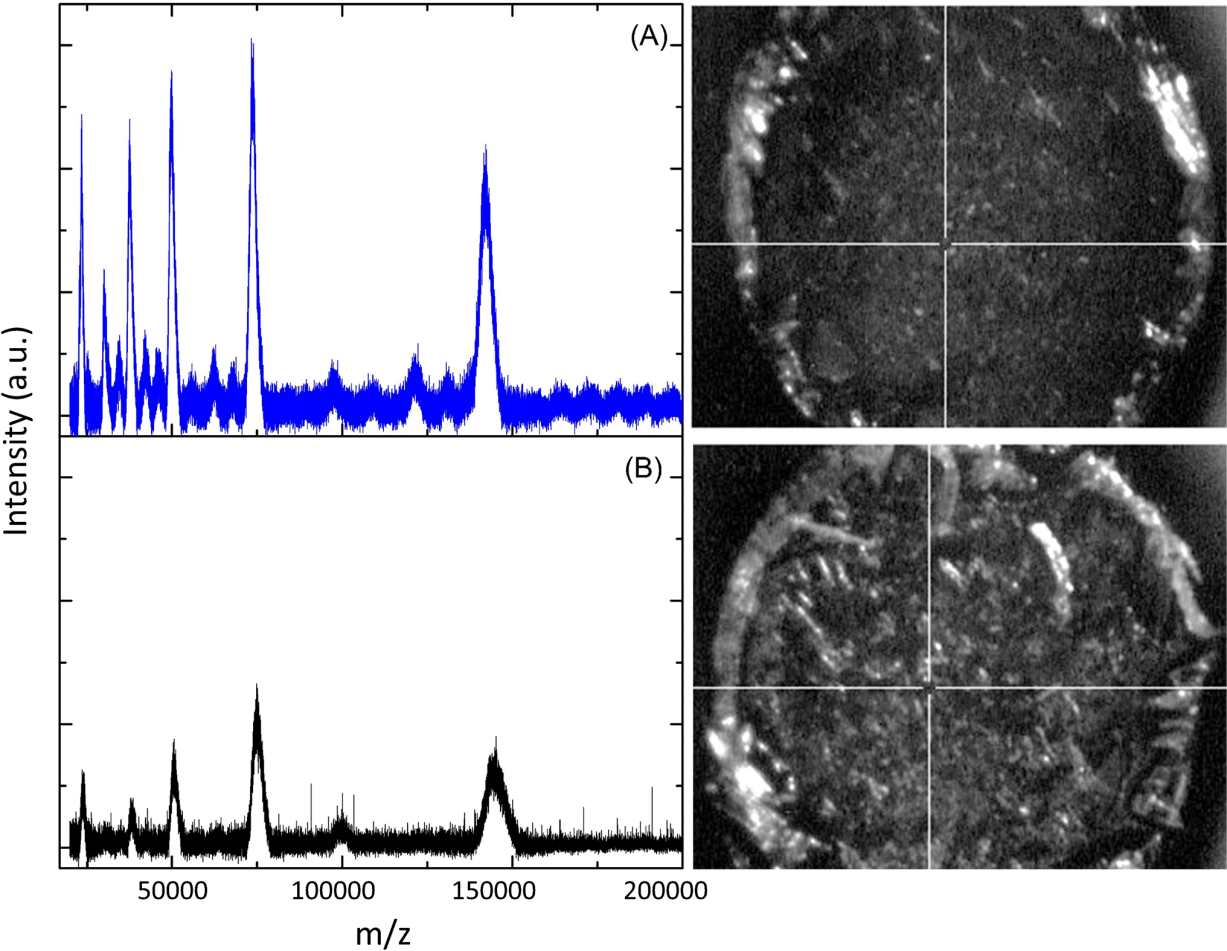
MS spectra of IgG (100 µg mL^−^^1^) measured in positive mode with (**A**) Cu_2_O PS@DHB prepared in ACN:0.1% TFA (30:70 v/v) and (**B**) Cu_2_O PS@DHB prepared in ACN:ultrapure water (30:70 v/v)

After setting the composition of the matrix, the effect of the presence or absence of TFA was evaluated to assist the ionization process. Figure 3 depicts the spectra of IgG (100 µg mL^−1^) under tested conditions. It was noted that acidification of the Cu_2_O PS@DHB allows a homogeneous distribution on the target spot in comparison with the not acidified matrix, where a rough surface is generated with an apparent aggregation of particles (Fig. 3B). In the last case, a direct impact on the ionization of analytes was observed even when the IgG solution contained 0.1% TFA. Hence, the proposed matrix was prepared in ACN:0.1% TFA (30:70 v/v) to achieve satisfactory deposition and the subsequent higher sensitivity in the analysis.

Another critical factor evaluated was the laser intensity due to its influence on the desorption/ionization process. The laser was also operated at 40% and 60%, and the signal intensities were compared to those obtained at 80% of the total power. As expected, lower signal intensities were observed for all peaks in both cases; thus, laser power was set to 80% for subsequent analyses.

### Performance of the Cu_2_O PS@DHB as a multipurpose platform

#### Analysis of intact proteins

**Fig. 4:**
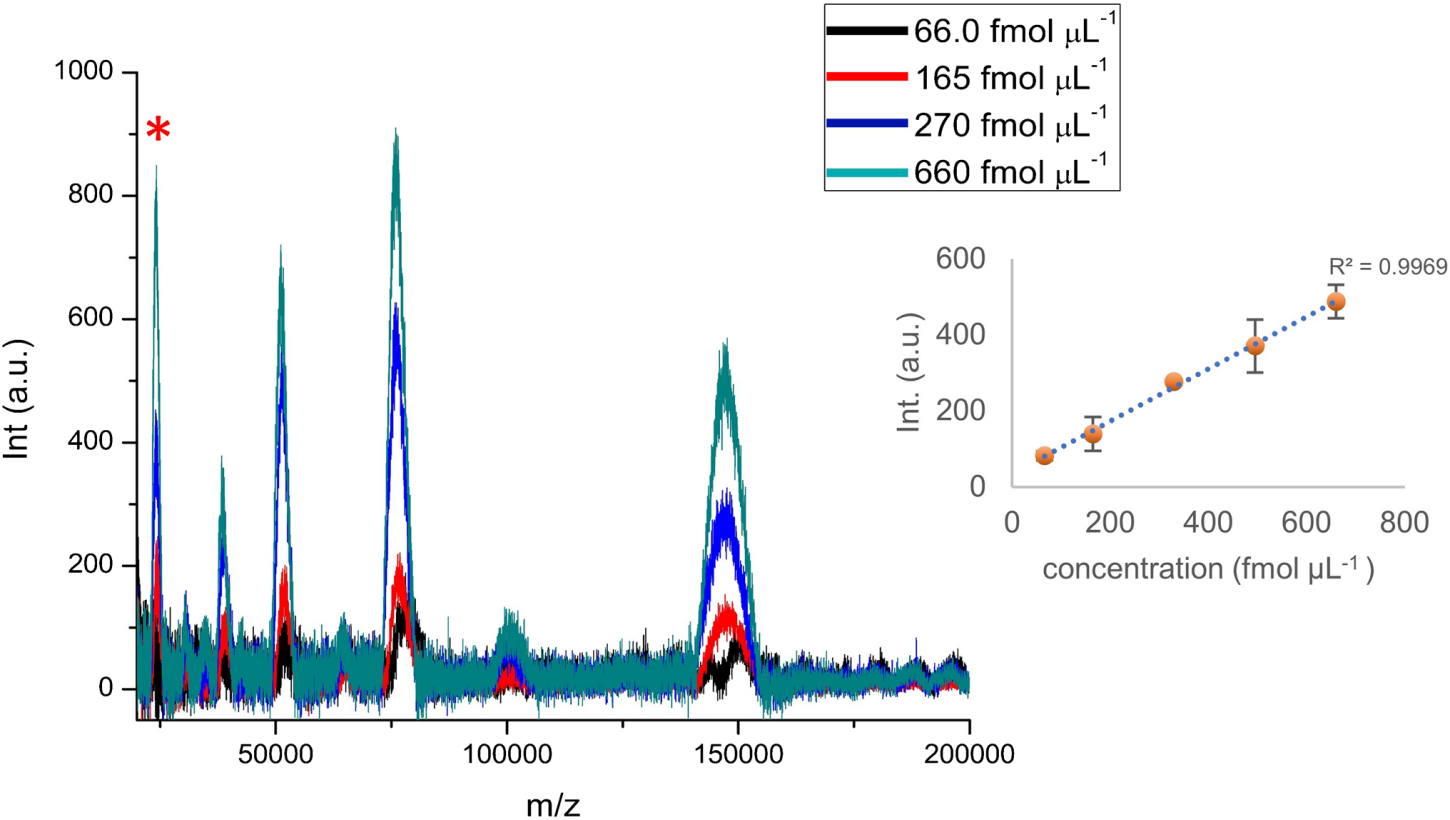
MS spectra of IgG standard under optimal conditions. Inset: correlation between intensity of [IgG + H]^+^ peak and concentration on spots (fmol µL^−^^1^), *n* = 5

Under optimal conditions, singly and multiply charged ions were detected in the analysis of the target IgG, mainly associated to the charge states (+ 1), (+ 2), (+ 3), and (+ 4). According to previous reports, the peak at m/z 23,500, marked with an asterisk in Fig. 4, is attributed to the ion of two identical light chains of IgG [15, 16]. For the other MS peaks, a S/N ratio > 3 was considered as the signal detection criterion. In this case, good correlation was observed between the absolute intensity and the concentration of this large molecule over the 66.0–660 fmol µL^−1^ range. Moreover, satisfactory precision in the signal intensity was attained with Cu_2_O PS@DHB in comparison with that obtained with conventional DHB matrix at low IgG concentration (Fig. S4).

**Fig. 5:**
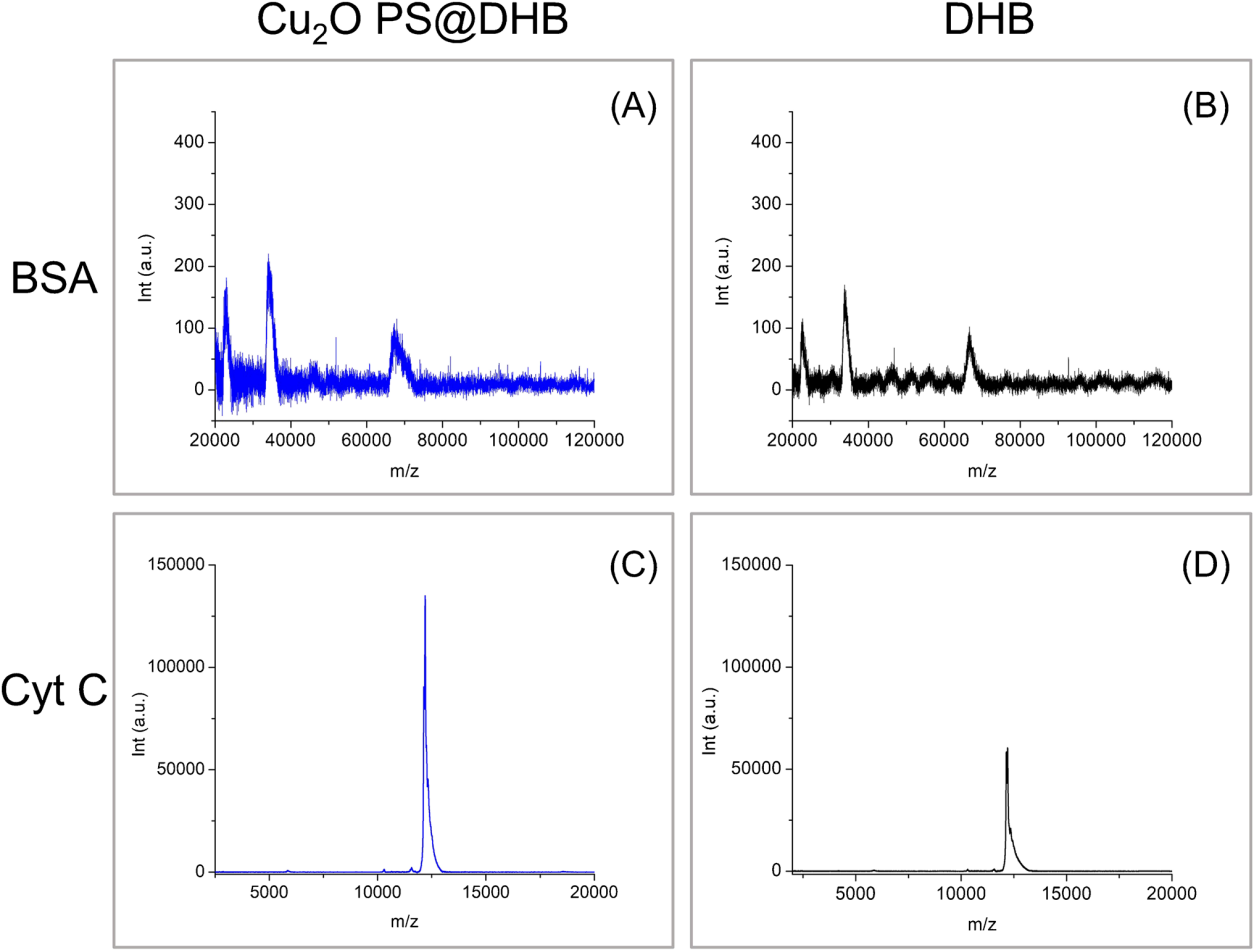
MS analysis of intact protein solutions at 10 µg mL^−1^ each. Spectra of BSA (**A**) and Cyt C (**C**) with Cu_2_O PS@DHB as matrix, and the comparison with the conventional DHB matrix (20 mg mL^−1^) in (**B**) and (**D**), respectively

Similarly, the analysis of intact BSA (66,430 Da) and Cyt C (12,384 Da) using the Cu_2_O PS@DHB matrix showed satisfactory results in terms of resolution of peaks and sensitivity in comparison to the conventional matrix (Fig. 5). As noted, three main peaks were detected for BSA, corresponding to the protonated adducts of the target protein, (+ 1), (+ 2), and (+ 3), while one high intensity peak is obtained for Cyt C. In this work, it is assumed that hydrogen bonds and electrostatic interactions, involving amino acid residues (e.g., tyrosine, tryptophan, lysine) in the proteins, could be the main driving forces in the binding to the Cu_2_O PS@DHB matrix, thus allowing more efficient energy transfer and ionization processes (Fig. S5).

It is worth to note that for BSA analysis, broadening of peaks was generated under optimal conditions, possibly attributed to a strong bioconjugation of the protein and Cu_2_O PS that could have an impact on the ionization and desorption of the analyte in comparison with the other intact proteins. Nonetheless, satisfactory signals were still obtained for BSA when applying the Cu_2_O PS@DHB matrix (~ 1.1 to 1.3-fold improvement in signal intensity) in comparison with the conventional matrix. Aiming to evaluate the BSA-particles interaction, UV–Vis spectroscopy studies were initially performed on Cu_2_O PS in contact with BSA and supported on glass slides. The Cu_2_O PS-BSA bioconjugation was confirmed by the changes in the optical response of the particles, where an increase in the absorbance and a slight blue shift on the LSPR position were observed due to the modifications on the surrounding environment (Fig. S6). As a counterpart, the variations in fluorescence signal were also tested for both, pure BSA molecules and the bioconjugate, prepared in the same conditions as above (Fig. S7). Upon interaction with the particles, the emission intensity of BSA decreases in comparison with its native signal. This partial quenching of the fluorescence is possible due to the adsorption of BSA molecules on Cu_2_O PS, preceded by the coordination with the metal through amino acid–containing fluorophore groups, such as tryptophan and tyrosine [23]. Besides, ATR-FTIR spectra were collected for particles before and after interaction with the protein, where no significant bands could be associated with any excess of reagents (Fig. S8). In this context, it is confirmed that the minimum amount of DHB used in the matrix does not modify the surface of particles to a great extent. Hence, protein-particle interaction could be mainly explained through association with the atoms on the surface of the Cu_2_O PS, in agreement with previous studies [23]. From the spectrum in Fig. S8, the typical amide I band of BSA is evidenced around 1644 cm^−1^ (C = O stretching) as expected for a protein with a secondary structure as a β-sheet (1637–1614 cm^−1^) [24]. The band at 1547 cm^−1^ can be attributed to amide II (C–N stretching coupled with N–H bending). The hydrophobic tail regions of BSA are assigned at 2850 and 2922 cm^−1^, representing symmetric CH_2_ (υ_s_CH_2_) and antisymmetric CH2 (υ_as_CH_2_) stretching bonds, respectively. It can be assumed that the interaction of BSA with Cu_2_O PS and its possible influence on the aggregation of the protein could be the reason for broader peaks in the MS spectrum.

#### Comparison of Cu_2_O PS@DHB with other platforms for analysis of proteins

The applicability of the proposed platform was compared with previous studies involving the analysis of intact Cyt C, IgG, and BSA [15–17, 25, 26]. Table 1 summarizes the concentrations determined using platforms containing micro- and nanomaterials with different compositions and morphology. It can be observed that results presented in this work meet those obtained by matrix-enhanced surface-assisted laser desorption/ionization based on a hybrid substrate, PAN/Nafion®/CNTs matrix, for IgG and even lower concentrations are detected for BSA [25]. Similarly, superior performance was obtained with the proposed Cu_2_O PS @DHB matrix in comparison with inorganic nano-substrates based on HgTe and TiN for IgG and BSA. In the case of Cyt C, obtained results were better than those attained with platinum nanosponges (Pt NSPs), although with limited sensitivity if compared with TiN nanopillars. In the latter, the higher performance is possibly attributed to the intrinsic characteristics and shape of pillars. These findings reinforce the concept that thermal and optical characteristics of the supporting material, and the chemical interactions between the surface and the analyte are key factors in the LDI process. Hence, it can be noted that the proposed Cu_2_O PS @DHB platform performed effectively as a simple analytical substrate for the studied proteins without requiring careful sample preparation and handling.

**Table 1:** Minimum analyzed concentrations (fmol µL^−1^) for intact proteins by the proposed Cu_2_O PS @ DHB and a comparison with those reported in the literature

Analyte	Current work	Reported	Micro/nanostructure-based LDI platform	Ref
IgG	66.0	66.0	PAN/Nafion®/CNTs matrix	[25]
		5000	HgTe nanoparticles	[16]
		5000	TiN nanopillar substrate attached onto ITO (indium tin oxide)	[15]
BSA	150	2000	HgTe nanoparticles	[16]
		300	PAN/Nafion®/CNTs matrix	[25]
Cyt C	807	500	Boron-doped carbon nanowalls (B-CNWs)	[17]
		200	HgTe nanoparticles	[16]
		50.0	TiN nanopillar substrate attached onto ITO (indium tin oxide)	[15]
		15000	Platinum nanosponges (Pt NSPs)	[26]

Moreover, satisfactory precision with RSD values between 0.90 and 1.08% was achieved for mass analysis by the presented methodology (Table S1), though a slight m/z ratio shifting to lower values is detected for the three proteins in comparison with the theoretical average masses, with a relative error between 0.04 and 0.70%. This might be attributed to the high laser power (80%) used in this work and the later possible fragmentation in the ion source and/or during acceleration of molecules [27]. Additionally, the performance of the proposed platform for BSA analysis was compared with the results provided in the literature when using an organic matrix based on ionic liquids [27]. A means *t*-test at 95% of confidence level was included and there were no observed statistically significant differences in molecular weights between values obtained by the proposed Cu_2_O PS @DHB and those previously obtained (|*t*_calculated_|= 0.51 < *t*_tabulated(0.05, 6)_ = 2.45), thus confirming the usefulness of the presented methodology.

#### Analysis of IgG digest

**Fig. 6:**
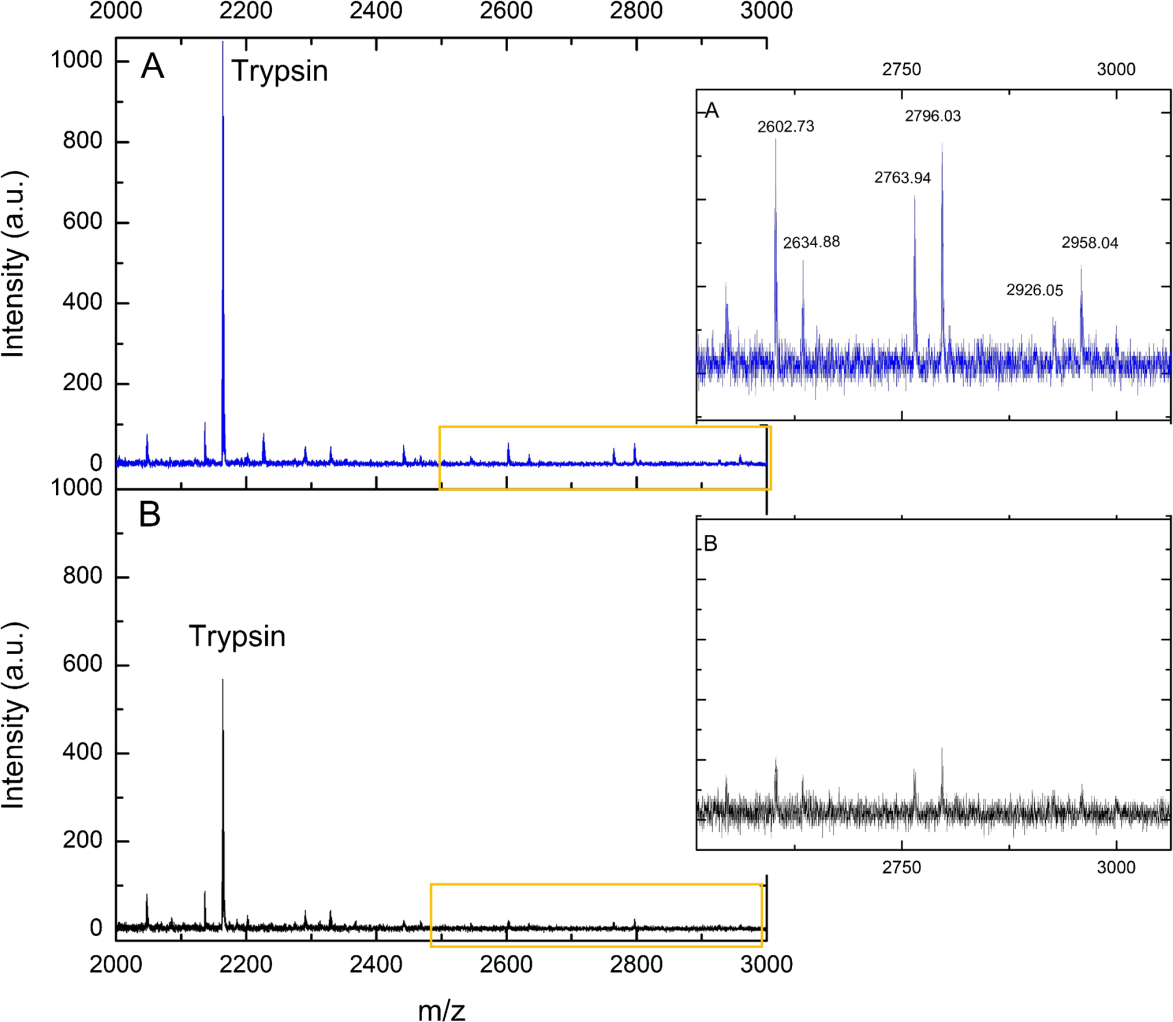
Direct analysis of IgG digest at 10 µg mL^−1^ by the proposed Cu_2_O PS @DHB (**A**) and DHB matrix, 20 mg mL^−^^1^ (**B**)

Along with the study of the intact proteins, the analysis of glycopeptides from IgG can be suitable both in diagnosing and treating diseases, such as cancer and degenerative and autoimmune/inflammatory diseases, and also in a better understanding of their progression [28]. To illustrate the applicability of the platform, Cu_2_O PS @DHB was tested for direct analysis of an unenriched IgG standard digest (10 µg mL^−1^), and glycopeptides were identified in the mass range between 2500 and 3000 Da (Fig. 6). Characteristic peaks with m/z ca. 2602, 2634, 2764, 2796, and 2958 showed satisfactory intensity signals at S/N > 3 with RSD% between 3.6 and 11.8% (*n* = 5). These peaks were referred to the most abundant glycopeptides from the protein in agreement with the literature [18, 29–31]. From these preliminary tests, it is clearly evidenced that the combination of Cu_2_O PS @DHB with certain enrichment procedures could improve the efficiency and selectivity in glycosylation studies.

## Conclusions

In this study, green synthesized copper oxide particles (Cu_2_O PS), simply obtained by microwave irradiation, were proposed for the first time for MALDI-MS analysis. It was demonstrated that particles exhibited good photostability and aqueous solubility, which improved their interaction with biomolecules. Moreover, considering the high ionization efficiency of the obtained particles and the easy co-crystallization with DHB, the combined Cu_2_O PS@DHB proved to be a good platform for the analysis of both intact proteins, with molecular weights up to 150,000 Da, and main glycopeptides (2500–3500 Da) from IgG digests. In this sense, the homogeneous distribution achieved on the spot surface results in high reproducibility for MS analysis in the extended mass range. Under optimized conditions, the analysis of IgG, BSA, and Cyt C provided similar results to that obtained with the existing analogues based on metal and metal oxide micro/nanoparticles and, in some cases, revealed a superior performance. Distinctively, the proposed user-friendly methodology was demonstrated to be a fast and sensitive alternative for detection of intact proteins with large size and complex structures, and also glycopeptides at very low concentration. From the exploratory analyses in this work, the combination of the Cu_2_O PS @DHB with appropriate enrichment procedures may be useful for future application in clinical and diagnostic research.

### Supplementary Information

Below is the link to the electronic supplementary material.

Supplementary file1 (PDF 777 KB)

## Data Availability

The data that support the findings of this study are available from the corresponding author at reasonable request.
