# Supplementary material for: User-friendly platform for analysis of high mass intact proteins and glycopeptides by laser desorption/ionization-mass spectrometry based on copper oxide particles
Source: Anal Bioanal Chem. 2023 Dec 8;416(4):861–72. doi: 10.1007/s00216-023-05072-0 (PMC10800303; doi:10.1007/s00216-023-05072-0)
Supplement: Supplementary file 1 — Supplementary file1 (PDF 777 KB) [file 216_2023_5072_MOESM1_ESM.pdf]

Supplementary material

User-friendly platform for analysis of high mass intact proteins and glycopeptides by laser desorption/ionization-mass spectrometry based on copper oxide particles

Valeria Springer[1], Yuye Zhou[2], Ángela Y. Aguilera[1], Åsa Emmer[2*]

[1] INQUISUR- Departamento de Química, Universidad Nacional del Sur (UNS)-CONICET, Av. Alem 1253, Bahía Blanca, Buenos Aires, Argentina

[2] KTH Royal Institute of Technology, School of Engineering Sciences in Chemistry, Biotechnology and Health, Department of Chemistry, Analytical Chemistry, 100 44 Stockholm, Sweden

*Corresponding author: aae@kth.se

Content

Figure S1. Pictures of spots after mixing IgG (100 µg mL^{-1}) with matrices (A) DHB and (B) Cu_2O PS@DHB

Figure S2. MALDI-MS spectra of (A) Cu_2O PS@DHB and (B) conventional DHB, 20 mg mL^{-1}

Figure S3. MALDI-MS spectra of IgG, 100 µg mL^{-1} (330 fmol applied on the spot) measured in the positive ion mode with matrices: (A) DHB, 20 mg mL^{-1} (B) DHB, 10 mg mL^{-1} and (C) 10 mg mL^{-1} DHB with 2.5 mg mL^{-1} Cu_2O PS

Figure S4. Average S/N values for charged ions of IgG (20 µg mL^{-1}) obtained using the proposed Cu_2O PS@DHB and the comparison with DHB matrix (n= 5). Bars indicate standard error values for each MS peak

Figure S5. Scheme of the possible interaction between proteins, through amino acid residues, and Cu_2O PS@DHB matrix. Orange and grey spheres represent copper and oxygen atoms in the particles, respectively

Figure S6. Cu_2O PS interaction with BSA (0.01 mg mL^{-1})

Figure S7. Fluorescence spectra of Cu_2O PS, BSA and Cu_2O PS–BSA bioconjugate at 25ºC (λex= 280 nm)

Figure S8. ATR-FTIR spectra of Cu_2O PS and Cu_2O PS–BSA bioconjugate

Table S1. Average molecular weights (Da) of singly charged molecular ions obtained with the Cu_2O PS@DHB platform and those found in the literature (Mreported), in comparison with the theoretical values (Mtheoretical)

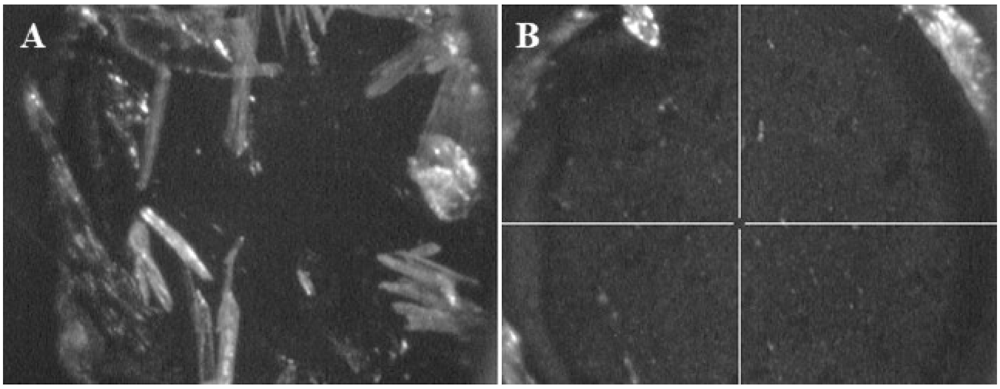

Fig. S1 Pictures of spots after mixing IgG (100 µg mL^{-1}) with matrices (A) DHB and (B) Cu_2O PS@DHB

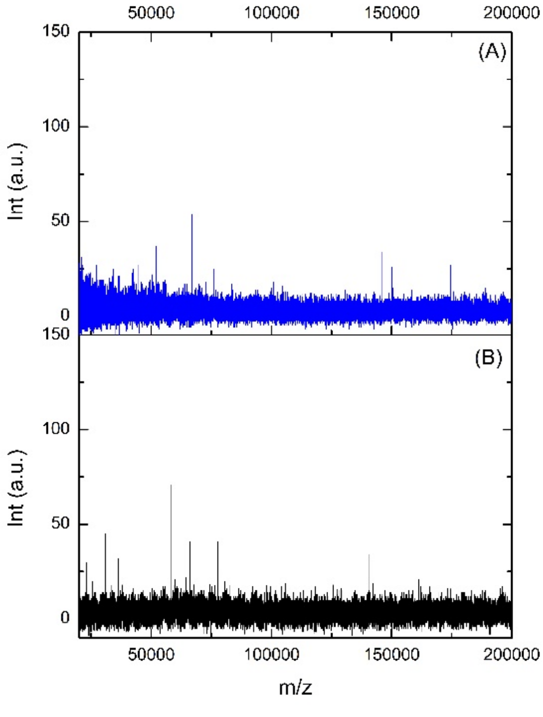

Fig. S2 MALDI-MS spectra of (A) Cu_2O PS@DHB and (B) conventional DHB, 20 mg mL^{-1}

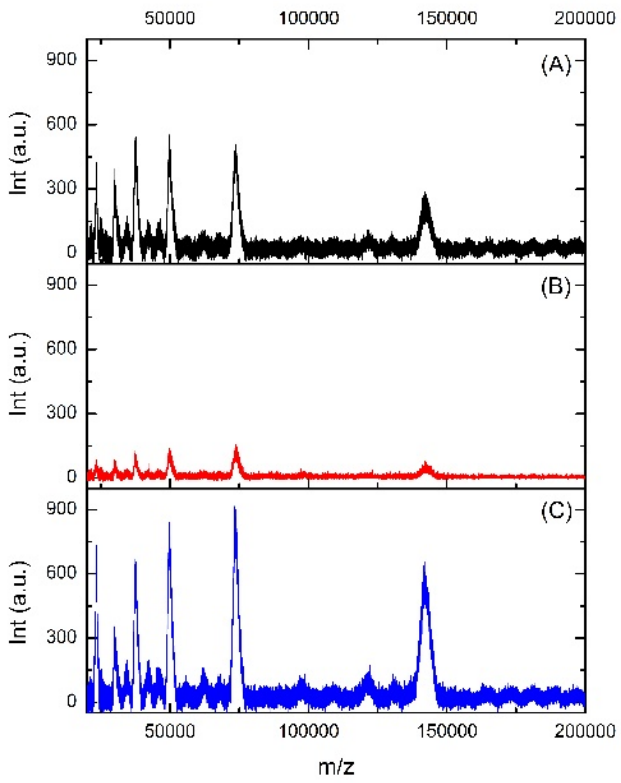

Fig. S3 MALDI-MS spectra of IgG, 100 µg mL^{-1} (330 fmol applied on the spot) measured in the positive ion mode with matrices: (A) DHB, 20 mg mL^{-1}, (B) DHB, 10 mg mL^{-1} and (C) 10 mg mL^{-1} DHB with 2.5 mg mL^{-1} Cu_2O PS

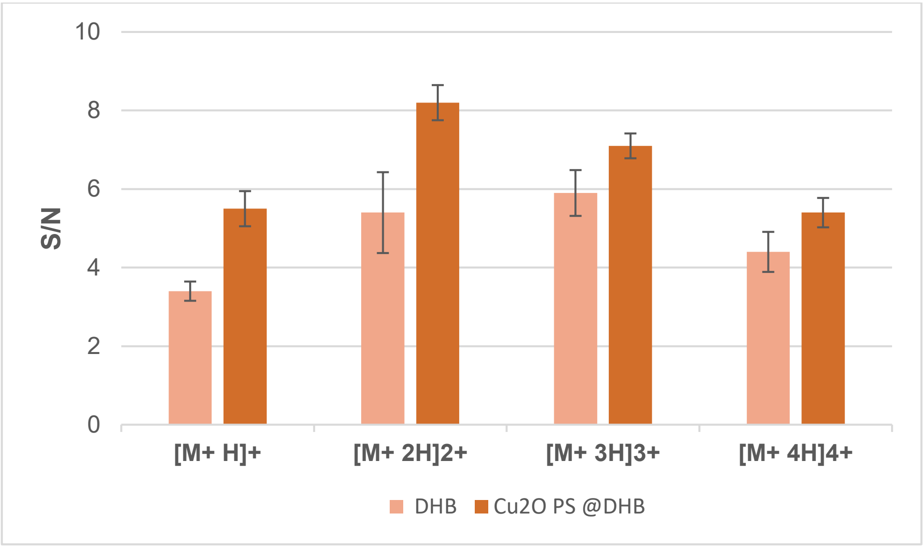

Fig. S4 Average S/N values for charged ions of IgG (20 µg mL^{-1}) obtained using the proposed Cu_2O PS@DHB and the comparison with DHB matrix (n= 5). Bars indicate standard error values for each MS peak

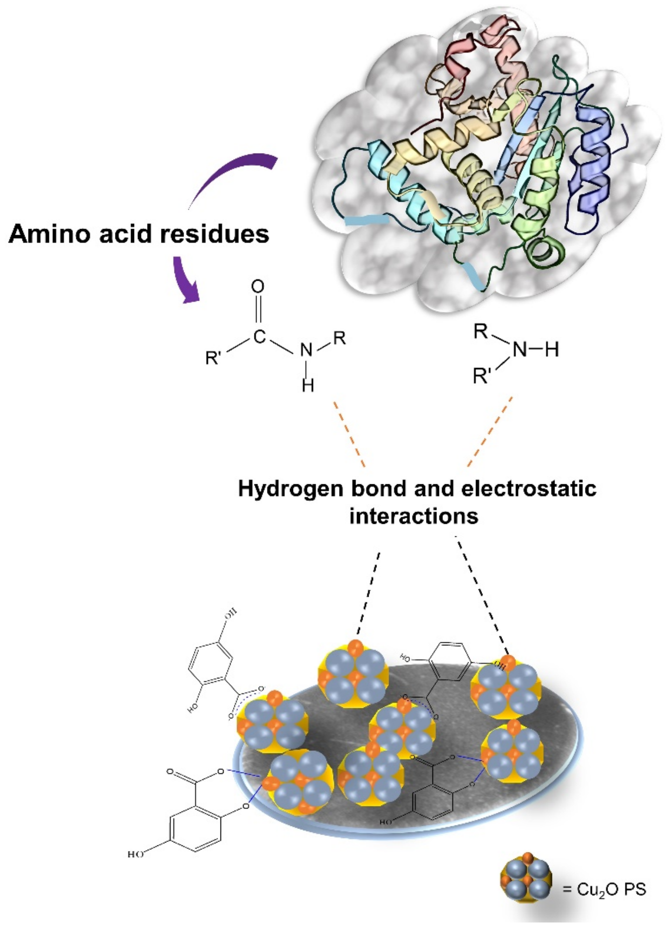

Fig. S5 Scheme of the possible interaction between proteins, through amino acid residues, and Cu_2O PS@DHB matrix. Orange and grey spheres represent copper and oxygen atoms in the particles, respectively

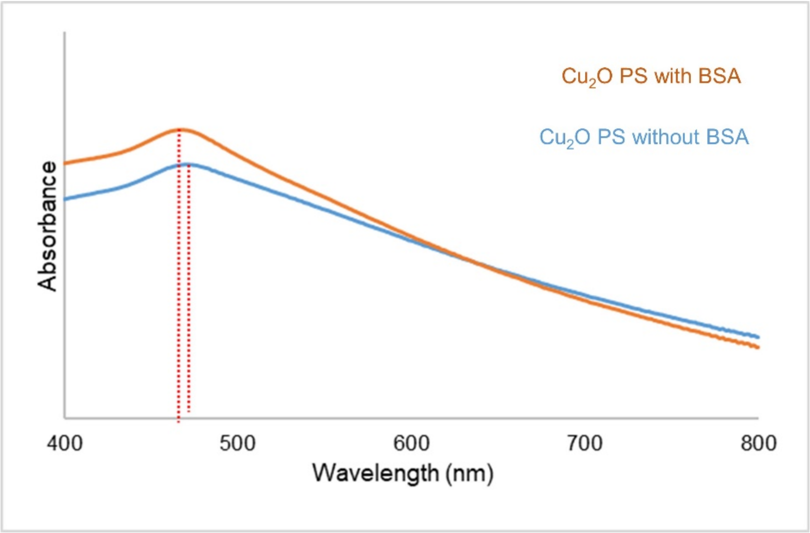

Fig. S6 Cu_2O PS interaction with BSA (0.01 mg mL^{-1})

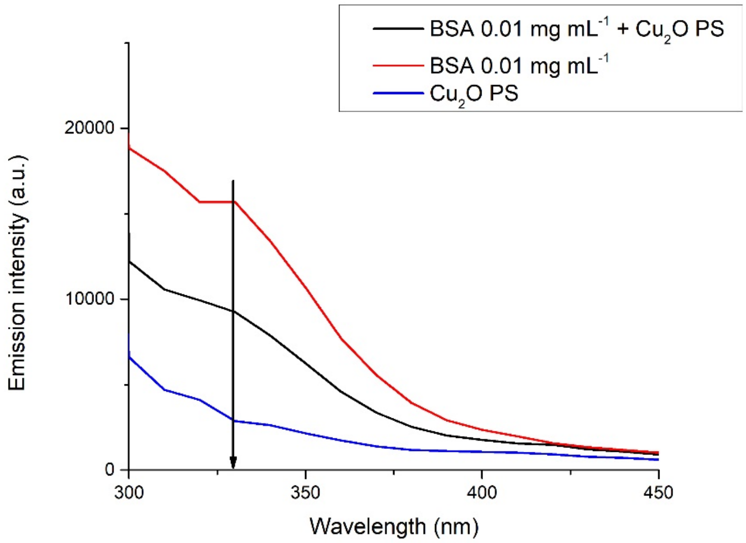

Fig. S7 Fluorescence spectra of Cu_2O PS, BSA and Cu_2O PS–BSA bioconjugate at 25ºC (λ_{ex}= 280 nm)

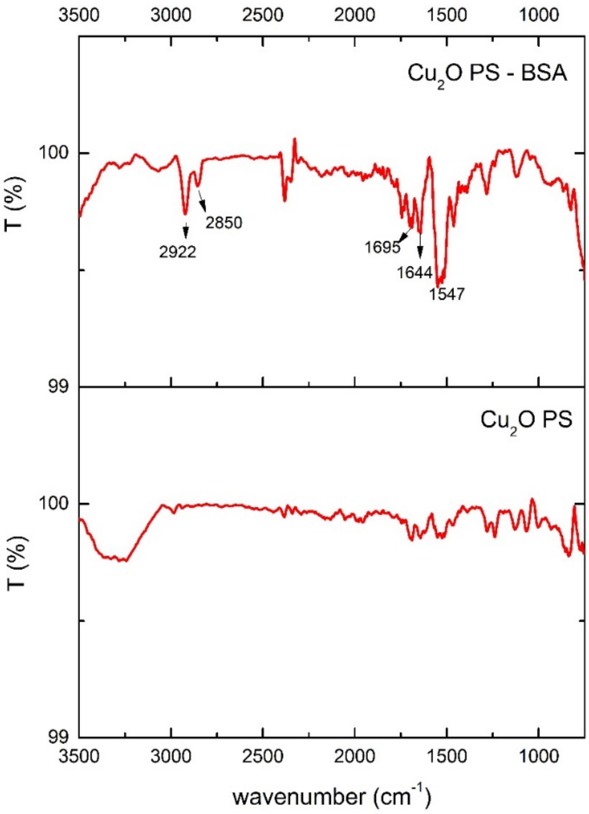

Fig. S8 ATR-FTIR spectra of Cu_2O PS and Cu_2O PS–BSA bioconjugate

Table S1. Average molecular weights (Da) of singly charged molecular ions obtained with the Cu_2O PS@DHB platform and those found in the literature ($M_{reported}$), in comparison with the theoretical values ($M_{theoretical}$)

Analyte	$M_{obtained} \pm s$*	$M_{theoretical}$	$M_{reported}$	Ref.
**IgG**	148988 ± 1445	150000	149000	[15]
**BSA**	66290 ± 716	66430	66512	[27]
**Cyt C**	12379 ± 111	12384	12385	[17]

* $M_{obtained}$ was calculated as an average of five replicates and s is the standard deviation
